# Viability fingerprint of glioblastoma cell lines: roles of mitotic, proliferative, and epigenetic targets

**DOI:** 10.1038/s41598-021-99630-0

**Published:** 2021-10-13

**Authors:** Darja Lavogina, Tõnis Laasfeld, Markus Vardja, Helen Lust, Jana Jaal

**Affiliations:** 1 Institute of Clinical Medicine, University of Tartu, L. Puusepa 8, 50406 Tartu, Estonia; 2 Institute of Chemistry, University of Tartu, Tartu, Estonia; 3 Department of Computer Science, University of Tartu, Tartu, Estonia; 4 Department of Radiotherapy and Oncological Therapy, Tartu University Hospital, Tartu, Estonia

**Keywords:** Cancer, Cell biology, Oncology

## Abstract

Despite the use of multimodal treatment combinations, the prognosis of glioblastoma (GB) is still poor. To prevent rapid tumor recurrence, targeted strategies for the treatment of GB are widely sought. Here, we compared the efficacy of focused modulation of a set of signaling pathways in two GB cell lines, U-251 MG and T98-G, using a panel of thirteen compounds targeting cell cycle progression, proliferation, epigenetic modifications, and DNA repair mechanism. In parallel, we tested combinations of these compounds with temozolomide and lomustine, the standard chemotherapy agents used in GB treatment. Two major trends were found: within individual compounds, the lowest IC_50_ values were exhibited by the Aurora kinase inhibitors, whereas in the case of mixtures, the addition of DNA methyltransferase 1 inhibitor azacytidine to lomustine proved the most beneficial. The efficacy of cell cycle-targeting compounds was further augmented by combination with radiation therapy using two different treatment regimes. The potency of azacytidine and lomustine mixtures was validated using a unique assay pipeline that utilizes automated imaging and machine learning-based data analysis algorithm for assessment of cell number and DNA damage extent. Based on our results, the combination of azacytidine and lomustine should be tested in GB clinical trials.

## Introduction

Glioblastoma (GB) is the most aggressive and lethal brain cancer in adults. Despite the use of multimodal treatment combinations, the prognosis of GB is still poor^1^. Since 1978, postoperative radiotherapy has been the mainstay of standard adjuvant treatment in GB^2^, which results in median overall survival of 12.1 months that can be extended to 14.6 months by administering temozolomide during and after radiotherapy^3^. In patients 70 years of age or younger, additional alternating electric field therapy can be utilized in combination with radiotherapy and temozolomide that prolongs median overall survival up to 20.9 months^4^.

Unfortunately, almost all GB patients die due to rapid tumor recurrence. Currently, no standard of care treatment is established when the disease progresses after radiotherapy and temozolomide. Out of possible treatment options that include repeat surgery and chemotherapy, a nitrosourea compound lomustine is most commonly used at disease recurrence. This approach is based on several phase II and III studies that have reported median overall survival times ranging from 5.6 to 9.8 months^5–9^. Moreover, previous studies have shown that re-irradiation provides temporary disease control in recurrent GB patients. A systematic review including 50 non-comparative studies of 2095 patients with recurrent GB who were treated with re-irradiation showed pooled 6- and 12-month overall survival rates of 73% and 36%^10^.

Since chemotherapy and radiotherapy result only in temporary disease stabilization in GB patients, additional, targeted strategies for the treatment of GB are widely sought. Among other options, selective modulation of epigenetic targets (e.g., DNA methyltransferases or histone deacetylases), cell cycle-related targets (e.g., mitotic protein kinases from the Aurora family), targets involved in DNA repair mechanisms and checkpoints (e.g., DNA-PK and Wee1) and multiple targets involved in proliferative and anti-apoptotic signaling pathways (e.g., somatostatin receptor and protein kinases Akt/PKB and CK2) is actively explored^11–13^. Although there are numerous reports investigating the efficiency of individual compounds, few studies have focused on a systematic comparison of roles of different pathways that define the characteristic viability profile of GB.

Here, we set out to compare the efficacy of targeted modulation of a set of signaling pathways in two GB cell lines, U-251 MG and T98-G. These cell lines differ in O6-methylguanine-DNA methyltransferase (MGMT) protein expression (T98-G is MGMT-positive and U-251 MG is MGMT-negative) and sensitivity to cytotoxic treatments (T98-G has shown high resistance to both radiotherapy and temozolomide^14^). By catalyzing demethylation of O6-methylguanine, MGMT contributes to the resistance of the cells to drugs that act via DNA alkylation; up to today, MGMT is the most studied prognostic biomarker in GB patients^15^.

For the assessment of targeted modulation in GB cells, thirteen individual compounds with focused selectivity profiles and combinations thereof with temozolomide and lomustine were screened using viability assay. After establishing the hotspots of interest, we also assessed the efficacy of one targeted drug candidate in combination with irradiation. Moreover, to characterize the effects of tested compounds and their combinations, fluorescence microscopy in fixed cells combined with the machine learning-based image processing pipeline was used.

## Results

### Viability assay

For establishing the viability fingerprint via modulation of the chosen signaling pathways, we defined a set of compounds of interest based on the relevant biological targets (see Supplementary Table S1). The candidates were chosen based on the available data regarding their potency, selectivity, and blood–brain barrier permeability; whenever possible, drugs that are currently used in clinics, and drug candidates that have advanced to the later stages of clinical trials were preferred^16–31^. The only compounds that have not been tested in clinical trials were somatostatin receptor (SSTR) antagonist CYN 154806^32,33^, and casein kinase 2 (CK2) inhibitor ARC-775^34^.

**Figure 1:**
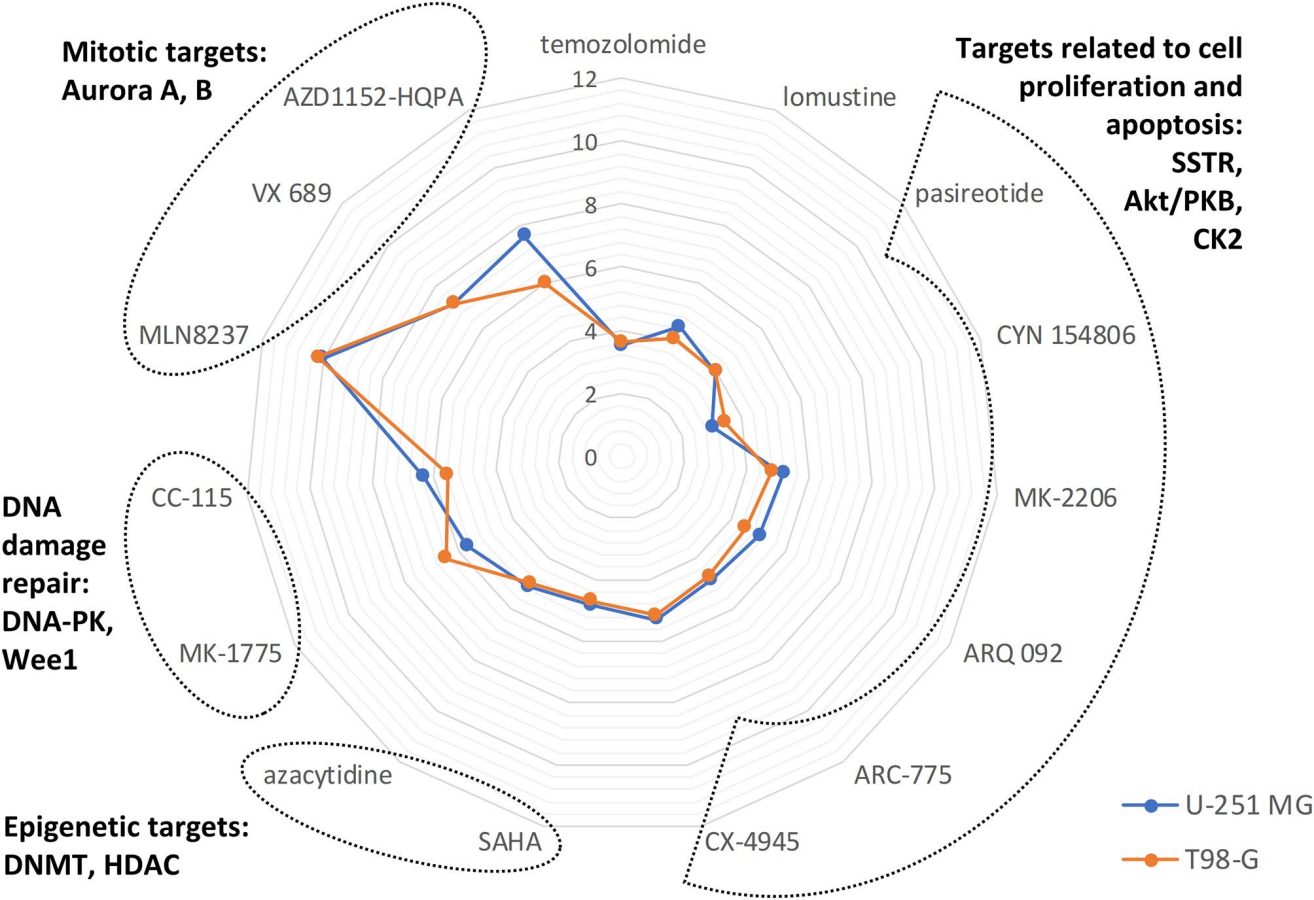
Viability fingerprint of GB cell lines following 48 h treatment. pIC_50_ values of individual compounds are shown for U-251 MG (blue) and T98-G (orange) cell lines. The further is the data point located from the center of the plot, the more efficient is the corresponding compound. The major biological targets of compounds are listed on the periphery.

The dose–response effect of individual compounds on viability of U-251 MG and T98-G cell lines following 48 h treatment is summarized in Table 1 and Fig. 1. Briefly, while temozolomide and lomustine showed moderate IC_50_ values in the submillimolar range, several targeted compounds featured remarkably lower IC_50_ values. Most of the targeted compounds were slightly more active in U-251 MG cells, except for SSTR antagonist CYN 154806 and Wee1 inhibitor MK-1775 for which lower IC_50_ values were observed in the T98-G line. Among the biological targets of interest, the most pronounced effect was observed in the case of mitotic Aurora kinases, as inhibitors of Aurora A/B featured submicromolar to subnanomolar efficiency in both cell lines. On the other hand, as indicated by the elevated bottom plateau of the dose–response curves (Table 1), these compounds could not trigger full mortality of cell population even at elevated concentrations, but rather abolished the proliferation of cells.

**Table 1:** Effect of individual compounds on viability of GBM cell lines (N ≥ 4).

Compound	U-251 MG			T98-G		
	pIC_50_ [CI]	IC_50_, µM	Bottom plateau, % [CI]	pIC_50_ [CI]	IC_50_, µM	Bottom plateau, % [CI]
Temozolomide	3.50 [3.63 to 3.37]	317	0 [− 5 to 4] (ns)	3.60 [3.73 to 3.48]	249	0 [− 5 to 5] (ns)
Lomustine	4.50 [4.57 to 4.43]	31.6	0 [− 3 to 4] (ns)	4.08 [4.15 to 4.00]	84.1	0 [− 5 to 4] (ns)
Pasireotide	4.02 [4.28 to 3.76]	96.4	0 [− 8 to 8] (ns)	4.00 [4.30 to 3.71]	99.3	0 [− 10 to 10] (ns)
CYN 154,806	3.02 [3.45 to 2.58]	962	0 [− 9 to 8] (ns)	3.44 [3.69 to 3.20]	360	0 [− 10 to 9] (ns)
MK-2206	5.17 [5.28 to 5.06]	6.82	1 [− 4 to 6] (ns)	4.77 [4.88 to 4.66]	17.1	− 6 [− 12 to 1] (ns)
ARQ 092	5.06 [5.17 to 4.96]	8.72	− 3 [− 8 to 3] (ns)	4.54 [4.67 to 4.42]	28.9	− 5 [− 13 to 2] (ns)
ARC-775	4.84 [4.96 to 4.73]	14.3	0 [− 8 to 9] (ns)	4.71 [4.84 to 4.57]	19.7	1 [− 9 to 10] (ns)
CX-4945	5.30 [5.41 to 5.19]	5.05	4 [− 5 to 11] (ns)	5.17 [5.30 to 5.04]	6.79	3 [− 7 to 12] (ns)
SAHA	4.83 [4.94 to 4.73]	14.8	− 1 [− 7 to 4] (ns)	4.70 [4.82 to 4.59]	20.0	− 4 [− 11 to 2] (ns)
Azacytidine	5.11 [5.24 to 5.00]	7.68	12 [7 to 17] (***)	4.99 [5.12 to 4.86]	10.3	6 [0 to 12] (ns)
MK-1775	5.69 [5.83 to 5.55]	2.06	27 [23 to 30] (***)	6.47 [6.60 to 6.34]	0.341	17 [14 to 21] (***)
CC-115	6.33 [6.43 to 6.24]	0.469	17 [14 to 19] (***)	5.57 [5.69 to 5.46]	2.68	16 [13 to 20] (***)
MLN8237	10.0 [10.4 to 9.69]	0.0000979	43 [39 to 48] (***)	10.2 [10.7 to 9.69]	0.0000669	56 [52 to 61] (***)
VX 689	7.22 [7.53 to 6.93]	0.0597	49 [43 to 55] (***)	7.23 [7.59 to 6.87]	0.0589	54 [47 to 61] (***)
AZD1152-HQPA	7.63 [7.97 to 7.30]	0.0235	66 [62 to 70] (***)	6.00 [6.40 to 5.60]	1.01	67 [61 to 73] (***)

CI, 95% confidence interval; ns, not significant; pIC_50_, negative logarithm of the half maximal inhibitory concentration. The asterisks indicate the significance of grouped comparison of the dose–response bottom plateau value obtained for each treatment condition relative to zero (one-way ANOVA; ***P < 0.001; ns, not significant).

**Figure 2:**
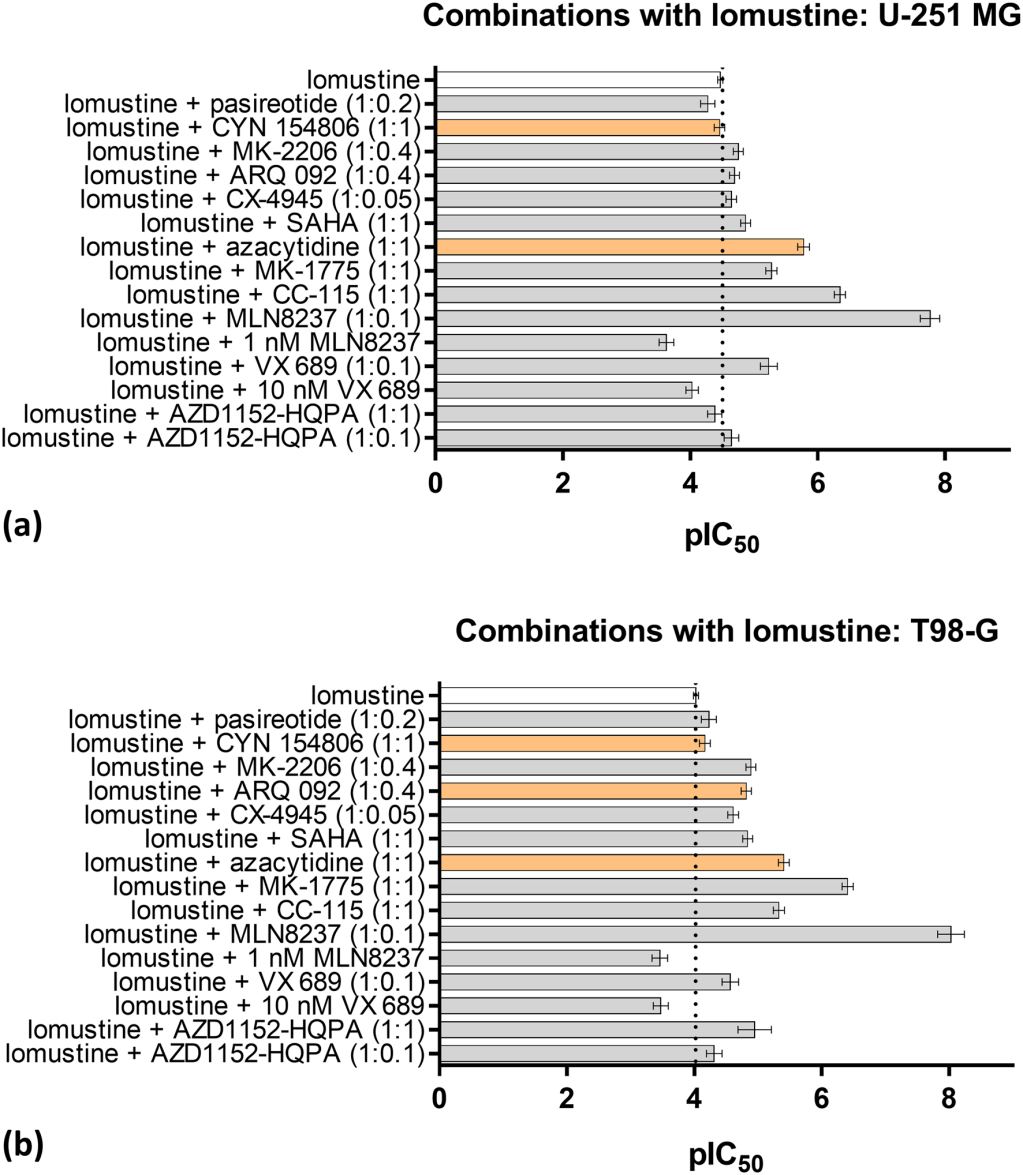
Effect of compound mixtures containing lomustine on the viability of GB cell lines. pIC_50_ values ± standard errors of mean are shown (N ≥ 4) for U-251 MG (**a**) or T98-G (**b**) following 48 h treatment. The further each column protrudes to the right, the more efficient is the corresponding mixture. In brackets, the molar ratios of components in each point of the dose–response curve are listed. The dotted vertical line shows the pIC_50_ value for lomustine alone. The columns colored in orange indicate mixtures for which the pIC_50_ value was significantly higher than the pIC_50_ value of either component within the given mixture (*P* < 0.05).

We next analyzed how the viability of GB cell lines was affected by mixtures composed of a targeted compound and a chemotherapy drug (either temozolomide or lomustine). The composition of mixtures was chosen based on the solubility of compounds in water, the comparability of the individual toxicities of compounds to those of temozolomide and lomustine, and the amount of available material. The mixtures tested and the corresponding dose–response pIC_50_ values are listed in Fig. 2 and Supplementary Figure S1. Briefly, in either cell line, several mixtures proved more efficient than the chemotherapy drugs alone, yet only a few mixtures were significantly more potent than the corresponding individual targeted compound included in the mixture (*P* < 0.05). Among the latter, the most outstanding were 1:1 molar mixtures of azacytidine with chemotherapy drugs, which showed consistently increased efficiency compared to the efficiencies of the corresponding single components. The shift of the dose–response curve in the case of azacytidine + lomustine (1:1) mixture is illustrated in Supplementary Figure S2.

### Radiation studies with MLN8237

To confirm the efficiency of Aurora A inhibitors that we observed in the viability studies, we proceeded to combined treatment of cell lines with MLN8237 and radiation (2 Gy or 4 Gy). Two treatment schemes were studied: in the first case (pre-treatment), 25 nM MLN8237 was added onto the cells 24 h prior to the irradiation, whereas in the second case (post-treatment), 25 nM MLN8237 was added onto the cells 2 h after the irradiation. In both cases, the viability of cells was analyzed 48 h after irradiation. As the controls, we also tested the effect of treatment with MLN8237 without irradiation, and the effect of irradiation without MLN8237 treatment.

**Figure 3:**
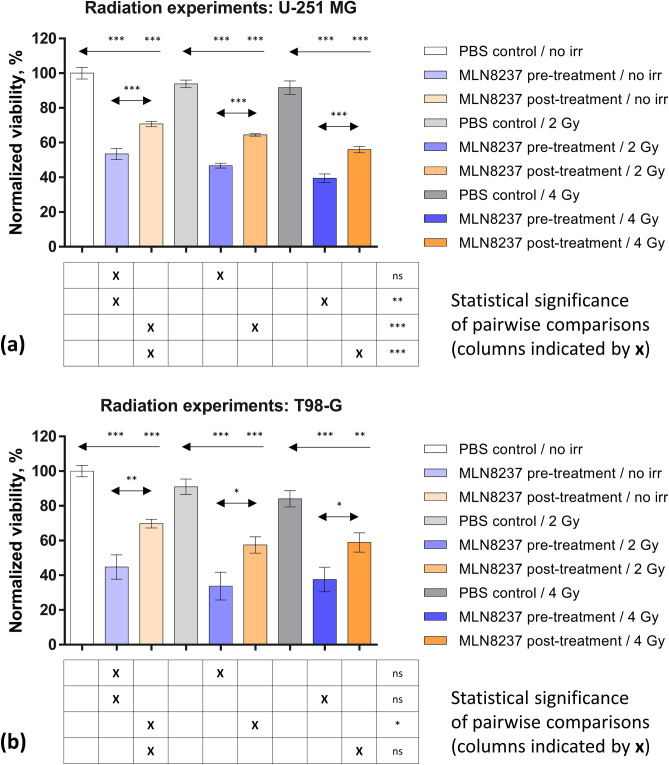
Potentiation of irradiation effect by MLN8237. Normalized viability values ± standard errors of mean are shown (N = 4). MLN8237 (final concentration of 25 nM) was added onto U-251 MG (**a**) or T98-G (b) cells either 24 h prior to irradiation (pre-treatment) or 2 h following the irradiation (post-treatment). Following irradiation, the cells were grown for a further 48 h. The asterisks above the top arrows in the graphs indicate the significance of grouped comparisons of each treatment condition to the PBS-treated cells on the same plate (one-way ANOVA; *** *P* < 0.001; ** *P* < 0.01). The asterisks above the middle set of arrows in the graphs indicate pairwise comparisons for the pre-treated and post-treated columns (unpaired t-test; *** *P* < 0.001; ** *P* < 0.01; * *P* < 0.05; ns, not significant). The grids below the graphs show pairwise comparisons (the compared columns are indicated with X) for the pre-treated or post-treated cells under different irradiation conditions (unpaired t-test; *** P < 0.001; ** P < 0.01; * P < 0.05; ns, not significant).

The results of radiation studies are summarized in Fig. 3 and Supplementary Figure S3. Briefly, within the timeframe of the assay, neither 2 Gy nor 4 Gy radiation alone caused a significant reduction of viability in GB cell lines relative to the non-irradiated control. Contrarily, treatment with MLN8237 caused significant reduction in cell viability (*P* < 0.01) in both cell lines for irradiated as well as non-irradiated plates, whereas the pre-treatment was consistently more efficient than the post-treatment (*P* < 0.05). The comparisons within the same treatment scheme revealed that in U-251 MG cells, co-treatment with MLN8237 and 4 Gy radiation was significantly more efficient than MLN8237 alone irrespective of the treatment scheme (*P* < 0.01); in T98-G cells, the combination of 2 Gy irradiation with MLN8237 post-treatment was more efficient than MLN8237 treatment alone according to the t-test (*P* < 0.05), but not according to the Mann–Whitney U-test (*P* = 0.07; Supplementary Figure S3).

### Immunofluorescence (IF) studies

To confirm the enhanced efficiency of lomustine + azacytidine mixture by an alternative methodology, we designed an assay enabling direct counting of cell number following treatment with individual compounds or their mixture. After 48 h treatment, the cells were fixed and stained with 4',6-diamidino-2-phenylindole (DAPI) to visualize nuclei; in parallel, to assess the DNA damaging effect of compounds and their mixture, immunofluorescent staining with antibody against phosphorylated Ser139 of histone H2AX (γH2AX) was carried out. For precise quantification of imaging data, an automated data analysis algorithm was developed that utilizes machine learning for detection of nuclei in the obtained images. The nuclei masks are then transferred to the γH2AX channel to establish the average intensity of antibody staining in each nucleus.

**Figure 4:**
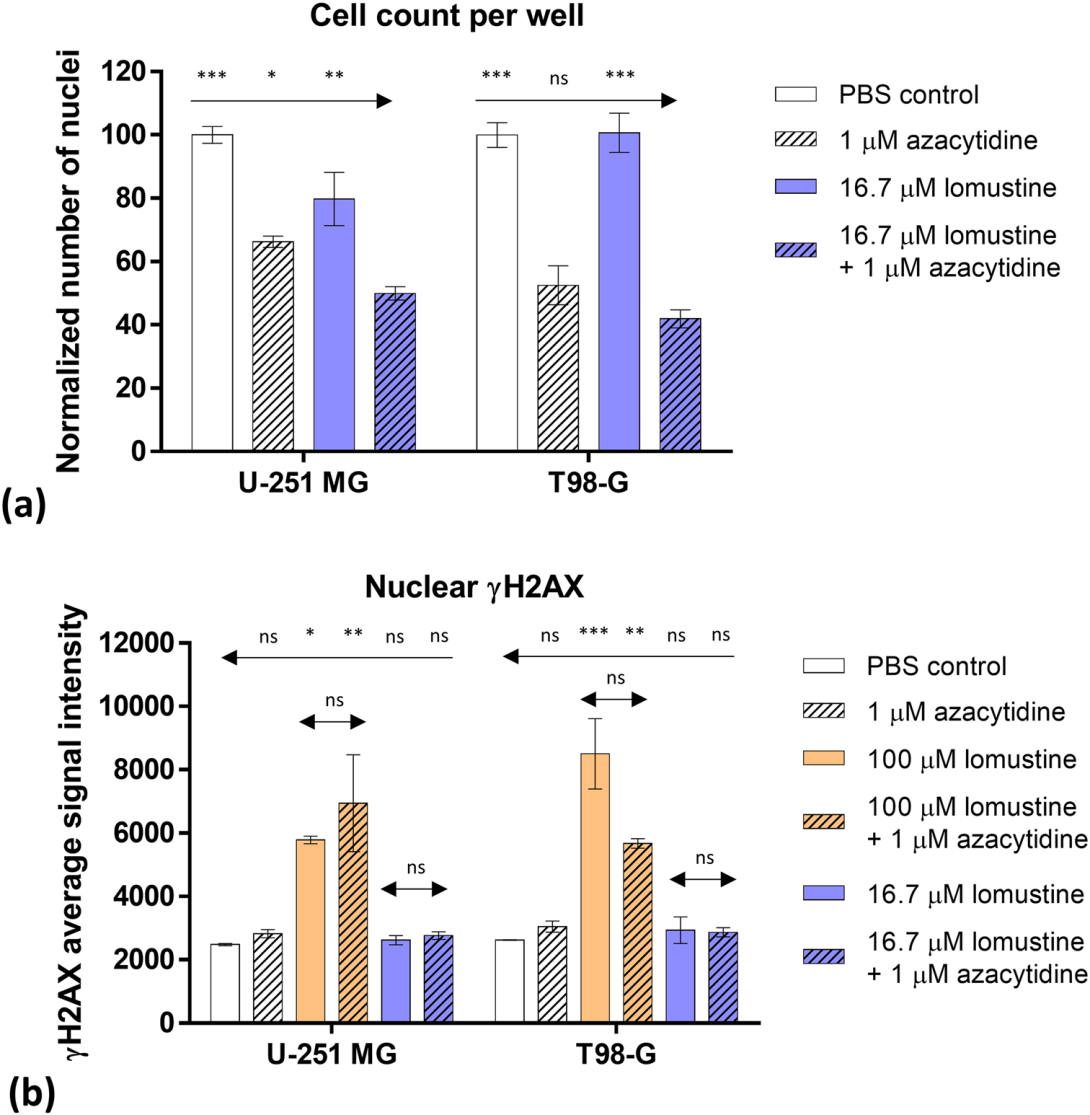
Confirmation of synergistic effect of lomustine and azacytidine in GB cell lines as determined by IF assay. (**a**) Effect of individual compounds and their mixture on the cell number following 48 h treatment. The graph shows the number of nuclei normalized to the PBS control for each individual experiment ± standard errors of mean (N = 3). The asterisks above the arrows in the graphs indicate the significance of grouped comparison of each treatment condition to the mixture-treated cells in the same cell line (one-way ANOVA; *** *P* < 0.001; ** *P* < 0.01; * *P* < 0.05; ns, not significant). (**b**) Effect of individual compounds and their mixtures on the γH2AX levels in the nucleus following 48 h treatment. The graph shows mean average γH2AX signal intensities ± standard errors of mean (N = 3). The asterisks above the arrows in the graphs indicate the significance of grouped comparison of each treatment condition to the PBS control in the same cell line (one-way ANOVA; ***P < 0.001; **P < 0.01; *P < 0.05; ns, not significant). The asterisks above the middle set of arrows in the graphs indicate pairwise comparisons for treatments with lomustine *vs* mixture (unpaired t-test; ns, not significant).

**Figure 5:**
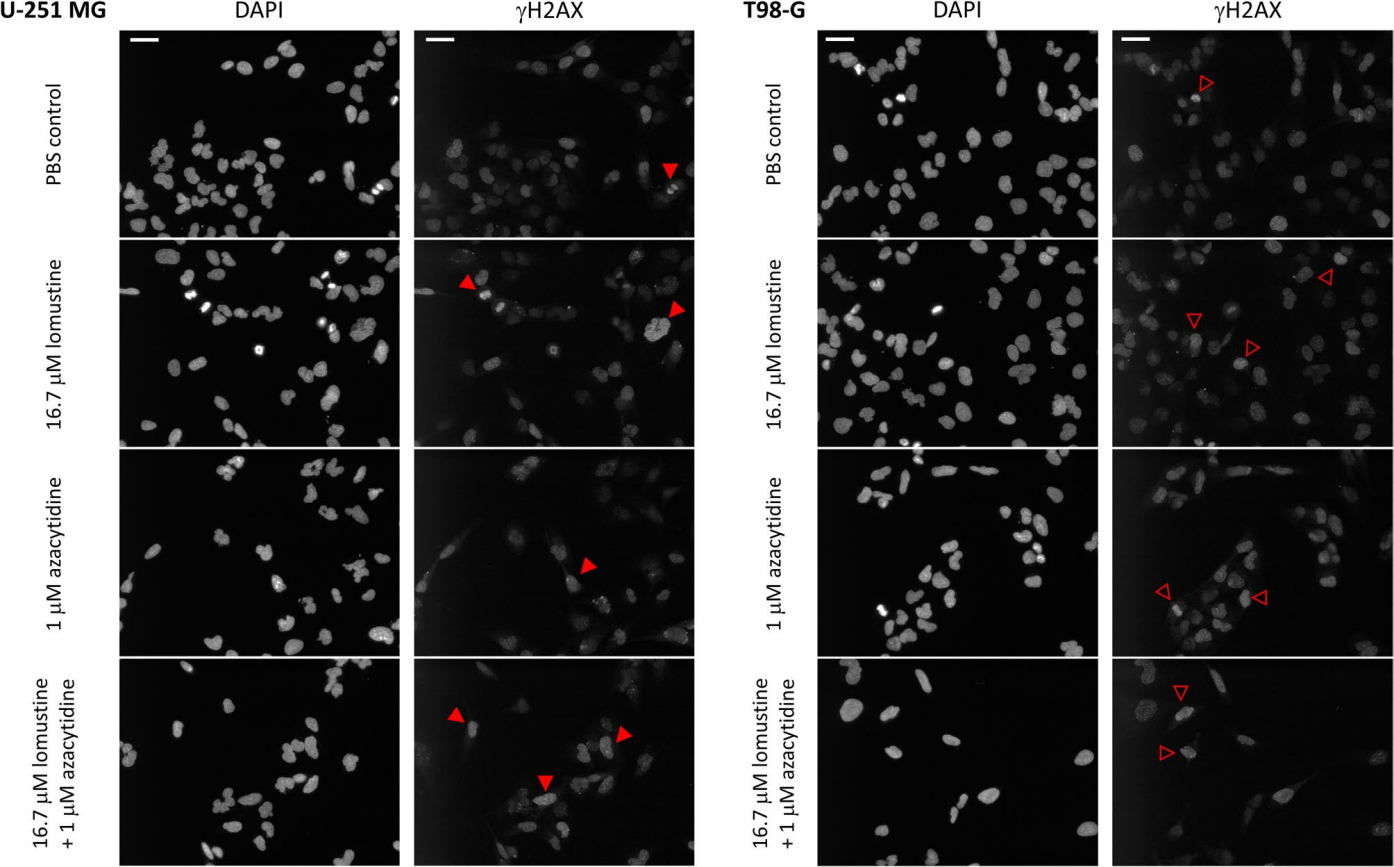
Representative microscopy images from IF assay in U-251 MG (left panel) and T98-G (right panel). The channels are indicated above the images and the treatment conditions (48 h) on the left. For better visualization, the brightness of images was increased by 40% and the contrast reduced by 40%; please note that the automated analysis of images was carried out using non-modified raw data. The images were collected within the same independent experiment. Scale bar (top left): 50 µm. Red arrowheads point to nuclei with strong γH2AX staining; empty arrowheads with red borders point to nuclei with medium levels of γH2AX staining.

The results of IF studies are summarized in Fig. 4 and Supplementary Figures S4-S5, and the representative microscopy images shown in Fig. 5 and Supplementary Figures S6-S7. The first set of experiments was carried out in both GB cell lines; for straightforward interpretation of the results, we utilized a constant concentration of azacytidine (1 µM, being thus 8–tenfold below the IC_50_ value of the individual compound according to viability studies) and dilution series of lomustine (sixfold dilutions starting from 100 µM concentration, in accordance with viability assay setup). Under these conditions, the number of nuclei that could be identified for the 100 µM lomustine (± 1 µM azacytidine) treatment was very low (Supplementary Figure S5). However, for the next dilution point, the total number of nuclei per well was sufficient to pool the data for 3 independent experiments (Fig. 5); it can be seen from Fig. 4a that the percentage of nuclei [relative to phosphate-buffered saline (PBS) treatment] was significantly higher in U-251 MG cells treated with 16.7 µM lomustine or 1 µM azacytidine alone, as compared to the corresponding mixture (*P* ≤ 0.05). In T98-G cells, the mixture was significantly more potent than lomustine alone (*P* ≤ 0.001), yet there was no statistically significant difference between the normalized number of nuclei in azacytidine *vs* mixture treatments.

On the other hand, while in either cell line, the signal intensity of nuclear γH2AX staining was significantly higher in case of treatment with 100 µM lomustine (± 1 µM azacytidine) as compared to the PBS control (*P* ≤ 0.05), there was no significant difference between 100 µM lomustine alone and its mixture with azacytidine. At lower concentrations of lomustine, γH2AX staining was not different from the PBS control (Figs. 4b, 5). The distribution of signals in individual nuclei in different treatments is shown in Supplementary Figure S4.

The second set of experiments (N = 3) was carried out in U-251 MG cell line only, and utilized dilution series of lomustine, azacytidine, or 1:1 molar mixture of the compounds (similarly to the viability assay). In this experiment, we calculated the dose–response pIC_50_ values for the effect of individual compounds and their mixture on the cell number. Supplementary Figure S5 shows that the pIC_50_ values of individual compounds are significantly lower than that of the 1:1 molar mixture (*P* ≤ 0.01), thus confirming that the mixture has higher efficiency than the individual compounds.

## Discussion

Due to poor therapeutic outcome and limited available treatment strategies for recurring cases, GB remains a challenging disease. While new biologically active compounds are sought for GB treatment, the repurposing of the already available drugs in novel combinations also offers an advantageous alternative. From the set of compounds chosen for our study, several compounds have been previously characterized in the context of GB. Yet to our knowledge, there are no reports on the systematic in vitro studies that directly compare effects of selective compounds targeting crucial cellular pathways on the viability of GB cell lines—and which we hence undertook here.

Out of all tested targeted therapies, modulation of somatostatin receptors both with somatostatin receptor agonist and antagonist was the least effective. Although previous studies in C6 rat glioma xenografts have shown that simultaneous activation of different somatostatin receptors (SSTRs) inhibits tumor cell proliferation through both direct cytostatic and antiangiogenic effects, we did not see much efficacy in human GB cell lines^35^. The latter observation is in line with previous in vitro studies that confirmed that somatostatin peptides do not exert anti-proliferative effects in human GB cells and probably do not have obvious value for GB therapy^36^.

One of the cellular pathways that has gained increasingly much attention in the context of GB is PI3K/Akt/mTOR. It has been shown that the gene encoding the phosphatase opposing activity of this pathway (PTEN) bears frequently inactivating mutations in GB^37^, thus necessitating the use of PI3K, Akt, or mTOR inhibitors to counteract the abnormally elevated activity of these kinases. The pan-Akt inhibitor MK-2206 was previously shown to enhance the effect of gefitinib in malignant glioma mouse model^28^, and act synergistically with temozolomide or irradiation in GB spheroids—yet not adherent cells^29^. However, these effects were consistently observed only at elevated concentrations of MK-2206 (0.5–10 µM), which was somewhat unexpected given that this allosteric inhibitor does not have to compete with a high intracellular concentration of ATP. In our viability assay (Table 1), both MK-2206, as well as another allosteric pan-Akt inhibitor ARQ 092^38,^ showed one- to two-digit micromolar IC_50_ values in U-251 MG and T98-G, respectively. Also, only a minimal increase in efficiency was observed upon combining ARQ 092 with lomustine (Fig. 2), which overall confirmed poor sensitivity of GB adherent cell lines to Akt inhibition.

Another potentially proto-oncogenic protein kinase that is abnormally active in GB is CK2, for which low-level amplifications were reported in one-third of glioblastomas, and which might also be indirectly connected to Akt signaling^39^. According to literature, the ATP-competitive inhibitor of CK2, CX-4945 suppressed the viability of GB cell lines (at 10 µM or higher concentrations when used individually, or at 1 µM concentration when combined with temozolomide), and was effective (individually or as a combination with temozolomide) in GB mouse models^26,27,39^. In our hands, the IC_50_ values for CX-4945 and its mixtures with temozolomide or lomustine also remained in the one- to two-digit micromolar range (Table 1, Fig. 2, Supplementary Figure S1), similarly to the IC_50_ values of the CK2 bisubstrate inhibitor ARC-775 (previously uncharacterized in GB context). It should however be noted that due to the limited solubility of CX-4945, its mixtures with chemotherapeutic agents contained high molar excess of the latter, thus improvement of solubility of CK2 inhibitors might be beneficial for more detailed studies of these compounds in the context of GB as well as other cancers.

Yet another family of protein kinases that is known to be aberrantly expressed in various cancers including GB is represented by the Aurora kinases, which are mapped on the intrinsically unstable regions of the genome^40^. Aurora A and B are indispensable for the progression of mitosis: Aurora A peaks at early mitosis, localizes to centrosomes and microtubules, and its overexpression overrides the mitotic checkpoint; Aurora B peaks at later phases of mitosis, and localizes to the kinetochores and midbody. Both kinases have been reported as the negative prognostic markers in GB^41,42^. Consistently, selective inhibitors of Aurora A [e.g., MLN8237 and VX 689^23,30,41,43^], Aurora B [e.g., AZD1152-HQPA^21,22^], as well as pan-Aurora inhibitors [e.g., AMG900, ZM 447439^44,45^] have been reported effective in GB cell lines as well as mouse models, when administered either as individual compounds, mixtures with temozolomide, or upon combination with irradiation.

In our hands, Aurora inhibitors, especially MLN8237 showed the highest efficiency among the tested individual compounds in both U-251 MG as well as T98-G (Table 1, Fig. 1); however, the viability of either cell line could not be reduced below 40% even at an elevated concentration of inhibitors, reflecting the fact that these compounds rather cause mitotic arrest than cell death per se. In mixtures of Aurora A inhibitors with chemotherapy agents, viability close to 0% was only observed at the highest concentration of temozolomide or lomustine, and fitting of dose–response curves indicated no improvement of IC_50_ values for the mixtures relative to the individual Aurora inhibitors (Fig. 2, Supplementary Figure S1). This indicates that the potential administration of Aurora inhibitors as individual drugs or as mixtures with chemotherapy agents may not be sufficient to achieve therapeutic success in GB treatment. On the other hand, the arrest of cancerous cells in mitosis can be advantageous when combined with the DNA-damaging effect of ionizing radiation^46^. In our studies (Fig. 3), we confirmed that treatment with a relatively low concentration of MLN8237 (25 nM) either prior to or following irradiation of GB cells significantly enhanced the effect of radiation (*P* ≤ 0.01) even within the short timeframe of the experiment (the viability of cells was measured 48 h after irradiation). Therefore, a combination of inhibitors of Aurora kinases (and, possibly, other cell cycle-related targets) with radiation holds great potential for GB treatment and should be studied in detail regarding the dosages (i.e., a lower amount of inhibitor might be sufficient as compared to the studies exploring the effect of individual compounds) and the treatment schemes to achieve full potential.

Interestingly, inhibition of protein kinase Wee1, which is also involved in cell cycle regulation as well as DNA damage recognition and repair pathways, was in our case significantly more efficient in T98-G than in the U-251 MG cell line (*P* ≤ 0.001; Fig. 1). In the case of Wee1 inhibitor MK-1775, earlier studies have reported the correlation of GB cell line resistance to the inhibitor with the elevated expression levels of Wee1^25^. Contrarily, CC-115, the dual inhibitor of DNA-PK and mTOR, was significantly more efficient in U-251 MG than in the T98-G cell line (*P* ≤ 0.001; Fig. 1).

The two cell lines used for our study were chosen based on the reported differences in MGMT expression: U-251 MG was shown to have poorly detectable MGMT protein levels, whereas T98-G expressed MGMT^47^. While increased MGMT protein levels are associated with a poor prognosis for chemotherapeutic treatment in GB^48,49^, we did not observe major differences between temozolomide efficiency in viability assay in U-251 MG versus T98-G. We presume that there might be two major reasons. First, the differences in effects of alkylating agents have previously been reported for U-251 MG versus T98-G in viability assays with at least 72 h treatment times^50,51^, or in the colony formation assays where the initial treatment times with compounds can be relatively short, yet the post-treatment period for monitoring recovery and growth of cells can last for weeks^52^. In our case, we performed viability assay directly following the treatment of cells, thus increasing the assay throughput, and enabling assessment of the initial, quick effects of compounds. Second, in our assay, temozolomide showed relatively high IC_50_ values in either cell line (Table 1); it is thus likely that in this range of IC_50_ values, the assay cannot distinguish sufficiently well between the subtle changes in cell viability following the short-term treatment. In case of lomustine as well as several other individual compounds, however, higher sensitivity of U-251 MG relative to T98-G could indeed be observed (Table 1, Fig. 1). Since the efficacy of cytotoxic chemotherapy and radiotherapy depends directly on DNA repair, the compounds and their combinations that augment the treatment-induced damage may also have a great potential in GB management. On the other hand, drug candidates that prove effective irrespective of the MGMT status of GB cells are also of utmost interest.

In the context of MGMT, the epigenetic regulation of gene expression have received a lot of attention in GB research: because methylation of the *MGMT* gene promoter is expected to reduce gene expression, the increased levels of *MGMT* promoter methylation are considered prognostic of the cancer responsiveness to treatment with cytotoxic cancer therapy^48,49^. Still, there has been conflicting evidence in the literature regarding the correlation between the MGMT methylation status and the enzyme expression level^53,54^. For instance, MGMT promoter methylation occurs in both U-251 MG and T98-G cell lines, whereas according to the enzyme expression level, U-251 MG is routinely classified as MGMT-negative and T98-G as MGMT-positive^52^. This is not surprising, as protein expression levels can be regulated via a variety of intracellular mechanisms, and the differences in ploidy of the cell lines of interest (diploid to triploid in case of U-251 MG, hyperpentaploid in case of T98-G) need also to be considered.

In our study, the epigenetic mechanisms were addressed via targeting DNA methyltransferases (DNMT) 1 and 3 (using azacytidine), and histone deacetylases (HDAC) 1 and 3 (using SAHA). Both classes of enzymes have been explored as drug targets in GB: while methylation of certain proteins can be beneficial for the survival of patients, hypermethylation of antiapoptotic genes can also occur^55^, and an imbalance between histone acyltransferases and HDACs has been reported for this disease^56^. The FDA-approved HDAC inhibitor SAHA has been studied in clinical trials for newly diagnosed GB (in combination with temozolomide or radiation) or recurrent GB^18,19^; in either case, the therapy had only modest efficacy. In our viability assays, SAHA also showed a very moderate, two-digit micromolar IC_50_ value—whether used individually (Table 1), or as a mixture with temozolomide or lomustine (Fig. 2, Supplementary Figure S1).

5-Azacytidine, on the other hand, has been studied individually in glioma and GB xenografts^16,17^, and in patients with recurrent glioma bearing mutations of isocitrate dehydrogenase genes^57^, as the latter frequently exhibit DNA hypermethylation. Despite stratification of patients, no conclusive results regarding the efficiency of azacytidine monotherapy could be achieved^57^. In our viability assay with resazurin, 48 h treatment with azacytidine resulted in 8 µM and 10 µM IC_50_ values in U-251 MG and T98-G, respectively (Table 1). Upon combining azacytidine with temozolomide or lomustine, the viability IC_50_ values were lowered fourfold in U-251 MG and threefold in T98-G relative to azacytidine alone; relative to lomustine alone, the 1:1 mixture of lomustine and azacytidine was over 20-fold more potent (Fig. 2 and Supplementary Figure S2). Such enhancement of lomustine potency in both cell lines was remarkable, as we initially envisioned a risk of boosting U-251 MG resistance to lomustine upon inhibition of DNMT via possible increase in MGMT levels. Outside of the GB context, there are reports in the literature regarding the re-expression of MGMT upon application of azacytidine^58–60^. It is likely, however, that even if azacytidine-triggered demethylation results in an increase of MGMT expression, the net effect of the compound on cells will rather be dictated by the dramatic changes in the expression of multiple genes, including cell cycle regulators and proapoptotic factors such as CDKN2B, GADD45γ and FHIT^61–63^. Overall, this issue requires further detailed inspection in both in vitro and in vivo models of GB.

Within our current study, the gain in efficiency of lomustine + azacytidine mixtures as compared to the individual components was further confirmed using a different methodology. We developed a unique assay pipeline that utilizes automated imaging and machine learning-based data analysis algorithm for assessment of cell number following 48 h treatment and fixation of cells.

Machine learning approaches have become useful tools for cellular and biomedical image analysis and are used not only in academia but also for regular histological and pathological diagnostics including cancer diagnosis^64,65^. More specifically, numerous attempts have been made for nuclei detection from fluorescence images using various software solutions^66–70^. Although deep-learning techniques usually offer the highest quality in controlled conditions, it is possible that the once trained deep-learning networks lose some performance when introduced to imaging data from a different microscope, magnification and cell line (commonly known as the domain shift). Therefore, for solving tasks where a relatively low number of manual annotations and comparatively simpler machine learning solutions can provide sufficiently high-quality results, the choice of the machine learning tool comes down to usability, clarity of user interfaces and flexibility of integration with other analysis tools. As the Ilastik software^71^ fulfils these criteria, allows training models with sufficient quality for nuclei detection, requires no special expertise in machine learning and has good documentation, it was chosen as a basis for nuclei detection in this study. Although the Ilastik model in this study was specifically trained for detecting nuclei from GB cell lines, retraining or generating models for other specific cell lines or microscopy equipment can be done swiftly. In this case, we integrated Ilastik models with MembraneTools module of Aparecium software for image post-processing steps such as removal of too small objects, calculation of γH2AX channel intensity for each nucleus, and batch processing. However, depending on the need, batch processing can also be carried out directly in Ilastik or integrated with other bioimage analysis software, such as FIJI^72^, for post-processing.

According to the IF assay, even the mixture containing 17-fold molar excess of lomustine to azacytidine was significantly more potent (*P* ≤ 0.01) in reducing the number of U-251 MG and T98-G cells than the corresponding concentration of lomustine alone (Fig. 4a). In cells treated with lomustine + azacytidine mixtures, the γH2AX signal was not different from the signal observed for lomustine or azacytidine treatment alone (Fig. 4b), thus indicating that enhancement of lomustine potency by azacytidine occurs by other means than interference with DNA damage recognition or repair pathways.

The detailed study of cellular mechanisms affected by azacytidine and lomustine co-treatment will be addressed in our future studies. Furthermore, the knowledge regarding the delivery of azacytidine to the brain is currently limited, necessitating exhaustive planning of future experiments and clinical trials. Nevertheless, previous preclinical studies have confirmed that both intrathecal^73^ and intraperitoneal^74^ administration of 5-azacytidine achieves adequate central nervous system exposure. Clinically, azacytidine is being tested in trials that allow participation of advanced solid tumor patients with treated and stable brain metastases^75^. Furthermore, taking into consideration that conventional radiotherapy can increase BBB permeability of chemotherapeutic drugs^76^, it is reasonable to believe that azacytidine reaches the brain tissue of glioblastoma patients—since almost all of them have received radiotherapy as their initial treatment, or as additional therapy in recurrent disease.

## Conclusions

*In corpora*, these findings thus point to the high potential of combined therapy with lomustine and azacytidine in patients with recurrent GB, where the dosage of azacytidine can be tuned to avoid possible side-effects on bone marrow. Importantly, as both lomustine and azacytidine have been long used in clinical practice, there is a wealth of data regarding the safety of either drug. As our future goal, we thus aim to conduct an academic phase II clinical study to evaluate the safety and efficacy of the combination of DNMT1 inhibitor azacytidine and lomustine in recurrent glioblastoma patients, whose life expectancy with current treatments is approximately 7.8 months.

## Materials and methods

### Chemicals and equipment

Human glioblastoma astrocytoma cell line U-251 MG and human glioblastoma cell line T98-G were from the American Type Culture Collection (ATCC; Manassas, VA, USA). Temozolomide, lomustine, MLN8237 (alisertib), VX 689 (MK-5108), AZD1152-HQPA (barasertib), MK-1775 (adavosertib), CC-115 and ARQ 092 were obtained from Selleckchem (Munich, Germany). Pasireotide (trifluoroacetate salt), MK-2206, azacytidine and suberanilohydroxamic acid (SAHA) were from Cayman Chemical (Ann Arbor, Michigan, United States); CYN 154806 (trifluoroacetate salt) was from Tocris / Bio-Techne (Bristol, UK); CX-4945 (silmitasertib) was from Synkinase (Shanghai, China). ARC-775 (prepared and purified according to the previously published procedures^34^) was a kind gift of Dr Asko Uri (Institute of Chemistry, University of Tartu, Estonia). The stock solutions of compounds were prepared in cell culture grade DMSO was from AppliChem (Darmstadt, Germany) and stored at − 20 °C; stock solutions of pasireotide and CYN 154806 were aliquoted for single use to avoid repeated freeze-thawing. The chemicals and labware required for cell culturing, viability assay and fluorescence microscopy are listed under the Supplementary Methods.

Fluorescence intensity and absorbance measurements were carried out with Synergy NEO or Cytation 5 multi-mode readers (both from Biotek; Winooski, VT, USA). During the radiation studies, the cells were exposed to 6 MV X-rays (Varian Truebeam 2.5). Fluorescence microscopy was carried out with Cytation 5 multi-mode reader using 20 × air objective. For DAPI, 365 nm LED and DAPI filter block were used; for Alexa Fluor® 568, 523 nm LED and RFP filter block were used.

### Treatment of cells for viability studies

The dose–response curves for individual compounds were performed in the 96-well format. U-251 MG or T98-G cells (passage number below 15) were seeded in growth medium onto the plate with a density of 3500 cells or 2500 cells per well, respectively (within the linear range of the method). After incubation for 24 h, the growth medium was exchanged, and dilution series of biologically active compounds in PBS were added onto the cells (the final volume of PBS relative to the fetal bovine serum-containing growth medium was 1/10). Based on solubility of compounds in water, the following final total concentrations were chosen: temozolomide, lomustine, MK-1775, CC-115, azacytidine, SAHA, CYN 154806—sixfold dilution starting from 100 µM; MK-2206, ARQ 092—sixfold dilution starting from 40 µM; pasireotide—sixfold dilution starting from 20 µM; ARC-775—sixfold dilution starting from 14 µM; MLN8237, VX 689, AZD1152-HQPA—sixfold dilution starting from 10 µM; CX-4945—sixfold dilution starting from 8.6 µM. An identical volume of PBS was added to the negative control (100% viability). The final volume per well was 100 µL, and the concentration of DMSO in the treated wells was ≤ 0.1% by volume; on each plate, each concentration of each compound was represented in duplicate.

The dose–response curves for the mixtures of compounds with temozolomide or lomustine were performed analogously. For most compounds (MK1775, CC-115, azacytidine, SAHA, CYN 154806, AZD1152-HQPA), 1:1 stoichiometric ratio was used (relative to temozolomide or lomustine) for the dilution series. Other stoichiometric ratios used were as follows (the first number corresponds to the molar amount of temozolomide or lomustine, and the second number to the molar amount of added chemical in each well of the dilution series): MK-2206, ARQ 092—1:0.4; pasireotide—1:0.2; MLN8237, VX 689, AZD1152-HQPA—1:0.1; CX-4945—1:0.05. For Aurora A inhibitors, the dose–response curves of temozolomide or lomustine were additionally measured at the fixed concentration of the added chemical (1 nM for MLN8237 and 10 nM for VX 689).

The cells were incubated with individual compounds or mixtures for 48 h, and viability assay was then carried out according to the previously published protocol^77^ (see details under the Supplementary Methods).

#### Radiation studies

The dose–response curves for individual compounds were performed in the 12-well format. U-251 MG or T98-G cells (passage number below 15) were seeded in the growth medium onto plate with the density of 10,000 cells or 5000 cells per well, respectively (within the linear range of the method). After incubation for 24 h, the growth medium was exchanged, and 25 nM MLN8237 (diluted in the growth medium) was added to some wells (4 replicates per plate; pre-treatment). After another 24 h, some plates were exposed to 2 Gy or 4 Gy of X-ray radiation; 2 h later, 25 nM MLN8237 (diluted in the growth medium) was added to some wells on the irradiated or non-irradiated plates (4 replicates per plate; post-treatment). An identical volume of PBS was added to the negative control (100% viability). The final volume per well was 1 mL, and the concentration of DMSO in the treated wells was 0.1% by volume. Following the post-treatment, the cells were grown for another 48 h, and viability assay was then carried out according to the previously published protocol^77^ (see details under the Supplementary Methods).

### IF and fluorescence microscopy

The treatment of cells was carried out as described above, with adjustment for the working volume (200 µL per well) and the number of seeded cells per well (4,000 cells in case of U-251 MG or 3000 cells in case of T98-G). Following 48 h treatment, the medium was removed, the cells were rinsed with PBS and fixed with cold methanol (15 min at − 20 °C). Next, the cells were washed with PBS and blocked for 1 h with 1% bovine serum albumin (BSA) in PBS (w/v) at room temperature; the overnight incubation at 4 °C with rabbit monoclonal IgG against γH2AX (Sigma-Aldrich, St Louis, MO, USA) followed (1:1000 dilution in 1% BSA/PBS). Afterwards, the cells were washed with 0.1% solution of Triton X-100 in PBS, and 3–6 h incubation at 4 °C with goat cross-adsorbed antibody against rabbit IgG (H + L) conjugated with Alexa Fluor® 568 (Invitrogen; Eugene, OR, USA) was carried out (1:1000 dilution in 1% BSA/PBS). The washing procedure was repeated, followed by 5 min staining with 300 nM solution of the nuclear stain DAPI (Invitrogen, Eugene, OR, USA) in PBS. Finally, the cells were washed with PBS and stored in PBS at 4 °C until imaging.

The imaging was performed in the automated mode; 25 images per well were taken and DAPI channel was used for autofocusing. The imaging settings (LED intensity/signal integration time/detector gain) were as follows: for DAPI, 4/104/5; for Alexa Fluor® 568, 6/252/19. The examples of strong nuclear staining indicating the suitability of the γH2AX antibody for the assay are presented in Supplementary Figure S7.

### Automated image analysis

For automated image analysis, a total of 11 images from DAPI channel were chosen for the training set containing images from multiple sequential focal planes to increase the algorithmic robustness against minor focusing errors. Ilastik software version 1.3.3b2 was used for all annotations and model training^71^. In the model, six features were used: Gaussian smoothing (σ_0_ value of 0.3 pixels), Laplacian of Gaussian, Gaussian gradient magnitude, difference of Gaussians, structure tensor eigenvalues and Hessian of Gaussian eigenvalues (all the features utilized σ_1–8_ values of 0.7, 1, 1.6, 3.5, 5, 10, 20 and 35 pixels). The pixels were classified into either the nuclei or background classes and all annotations were performed in Live Update mode. Model quality was incrementally increased by adding additional annotations to areas where the pixel misclassification was the highest. In most cases, these areas were located near the nucleus edge. The classification probability maps were binarized by using the probability threshold of 0.5. Nuclei with an area smaller than 200 pixels (20.8 µm^2^) were removed, as such objects were considered to be imaging or fixation artifacts. The trained model is available at https://gpcr.ut.ee/aparecium.html. The pipeline robustness against detection of possible microscopy artifacts was checked and confirmed visually. Quality metrics for the algorithm (see details in the Supplementary Table S2) were determined to be as follows: F1 score = 0.94, precision = 0.91, recall = 0.97, Matthews correlation coefficient (MCC) = 0.96. All quality metrics were thus sufficiently close to the perfect detection indicating that the image analysis pipeline was fit for purpose. Batch processing was carried out using the modified version of the MembraneTools module of Aparecium 2.0 software^78^. The software is available at https://gpcr.ut.ee/aparecium.html. Based on the location of the nuclear pixels, the objects were segmented into individual nuclei by connected component analysis. The average and total intensities of the individual nuclei were calculated for each image in both DAPI and γH2AX imaging channels. The data with average intensities were exported to comma-separated values (CSV) format and used for further statistical analysis.

### Statistical analysis

For data analysis, GraphPad Prism 6 (San Diego, CA, USA) and Excel 2016 (Microsoft Office 365; Redmond, WA, USA) were used. In the case of all methods, at least three independent experiments were performed. Throughout the study, the grouped comparisons were carried out using 1-way ANOVA with Dunnett’s test for multiple comparisons; unless indicated otherwise, the pairwise comparisons were carried out using the unpaired two-tailed t-test with Welch's correction. In all statistical tests, the significance of comparisons is indicated as follows: *** indicates *P* ≤ 0.001, ** indicates *P* ≤ 0.01, * indicates *P* ≤ 0.05. The details on the normalization of data and statistical analyses are provided under the Supplementary Methods.

## Supplementary Information

Supplementary Information.

## Data Availability

The datasets generated and analyzed during the current study are available from the corresponding author on reasonable request. The software and Ilastik model is available at 10.6084/m9.figshare.14749530.v1. The examples of non-modified microscopy images are available at https://figshare.com/s/35c7b3a19270592f881f.

## Abbreviations

DNMT: DNA methyltransferase
GB: Glioblastoma
SAHA: Suberanilohydroxamic acid
