# Supplementary material for: Viability fingerprint of glioblastoma cell lines: roles of mitotic, proliferative, and epigenetic targets
Source: Sci Rep. 2021 Oct 13;11:20338. doi: 10.1038/s41598-021-99630-0 (PMC8514540; doi:10.1038/s41598-021-99630-0)
Supplement: Supplementary file 1 — Supplementary Information. [file 41598_2021_99630_MOESM1_ESM.docx]

SUPPLEMENTARY DATA

**Viability fingerprint of glioblastoma cell lines: roles of mitotic, proliferative, and epigenetic targets**

Darja Lavogina,^1,2^ Tõnis Laasfeld,^2^ Markus Vardja,^3^ Helen Lust,^1^ Jana Jaal^1,3^

^1^Institute of Clinical Medicine, University of Tartu, Tartu, Estonia

^2^Institute of Chemistry, University of Tartu, Tartu, Estonia

^3^Department of Radiotherapy and Oncological Therapy, Tartu University Hospital, Tartu, Estonia

Table S1. Compounds and biological targets

| Compound | Target protein and effect | Cellular processes affected | Status in clinics |
| --- | --- | --- | --- |
| Pasireotide | Somatostatin receptor 1, 2, 3, 5; agonist | Cell proliferation | Approved for treatment of Cushing's disease in the US and EU |
| CYN 154806 | Somatostatin receptor 2; antagonist | Cell proliferation | Preclinical |
| MK-2206 | Protein kinase B; allosteric inhibitor | Cell proliferation | Clinical trials, Phase II |
| ARQ 092 | Protein kinase B; allosteric inhibitor | Cell proliferation | Clinical trials, Phase II |
| ARC-775 | Casein kinase 2; bisubstrate inhibitor (prodrug) | Cell proliferation | Preclinical |
| CX-4945 | Casein kinase 2; ATP-competitive inhibitor | Cell proliferation | Clinical trials, Phase II |
| SAHA | Histone deacetylase 1 and 3; inhibitor | Epigenetic modifications | Approved for treatment of cutaneous T cell lymphoma in the US |
| Azacytidine | DNA methyltransferase 1 and 3; inhibitor | Epigenetic modifications | Approved for treatment of myelodysplastic syndrome in the US and EU |
| MK-1775 | Protein kinase Wee1; ATP-competitive inhibitor | DNA repair, mitosis | Clinical trials, Phase II |
| CC-115 | Dual inhibitor of DNA-dependent protein kinase and mammalian target of rapamycin | DNA repair, cell proliferation | Clinical trials, Phase II |
| MLN8237 | Protein kinase Aurora A; ATP-competitive inhibitor | Mitosis | Clinical trials, Phase II |
| VX 689 | Protein kinase Aurora A; ATP-competitive inhibitor | Mitosis | Clinical trials, Phase I |
| AZD1152-HQPA | Protein kinase Aurora B, ATP-competitive inhibitor | Mitosis | Clinical trials, Phase I |

Table S2. Quality metrics for the automated image analysis algorithm

| Parameter and alternative names | Explanation | Range of values | Formula | Citation |
| --- | --- | --- | --- | --- |
| Precision; positive predictive value | In pixel classification, precision shows the ratio of true human annotated nuclear pixels and all pixels detected by the algorithm. | 0 (no predicted pixels are nuclei) to 1 (all predicted pixels are nuclei) | $\frac{TP}{TP+FP}$ | - |
| Recall; sensitivity; hit rate; true positive rate | Recall is the ratio of correctly detected nuclear pixels to the total number of true human annotated nuclear pixels. | 0 (no true nuclear pixels detected) to 1 (all nuclei pixels detected) | $\frac{TP}{TP+FN}$ | - |
| F1 score; Sørensen-Dice coefficient | F1 score and MCC serve as different kinds of weighted averages for precision and recall. | 0 (precision, recall, or both are 0) to 1 (both precision and recall are 1) | $2\cdot\frac{Precision\cdot Recall}{Precision+Recall}$ | ^1,2^ |
| Matthews correlation coefficient (MCC); phi coefficient; Yule phi coefficient |  | -1 (perfect anticorrelation) to 1 (perfect precision and recall) | $\frac{TP\cdot TN-FP\cdot FN}{\sqrt{(TP+FP)(TP+FN)(TN+FP)(TN+FN)}}$ | ^3^ |

Abbreviations: TP – true positive prediction; TN – true negative prediction; FP – false positive prediction; FN – false negative prediction

Figure S1. Effect of mixtures containing temozolomide on viability of GBM cell lines. pIC_50_ values ± standard errors of mean are shown (N ≥ 4) for U-251 MG (a) or T98-G (b). The further each column protrudes to the right, the more efficient is the corresponding mixture. In brackets, the molar ratios of components in each point of the dose-response curve are listed. The dotted vertical line shows the pIC_50_ value for temozolomide alone. The columns colored in orange indicate mixtures for which the pIC_50_ value was significantly higher than the pIC_50_ value of either component within the given mixture (P < 0.001).

Figure S2. Comparison of viability assay dose-response curves for lomustine, azacytidine, and 1:1 mixture of lomustine and azacytidine. Pooled normalized data ± standard error of mean is shown (N ≥ 4). MAX corresponds to 100% viability (PBS-treated control) and MIN to 0% viability (no cells seeded to the well).

Figure S3. Potentiation of irradiation effect by MLN8237 (corresponds to Figure 3 in the main text except for the statistical test used for the pairwise comparisons). Normalized viability values ± standard errors of mean are shown (N = 4). MLN8237 (final concentration of 25 nM) was added onto U-251 MG (a) or T98-G (b) cells either 24 h prior to irradiation (pre-treatment) or 2 h following the irradiation (post-treatment). Following irradiation, the cells were grown for further 48 h. The asterisks above the top arrows in the graphs indicate significance of grouped comparisons of each treatment condition to the PBS-treated cells on the same plate (one-way ANOVA; *** P < 0.001; ** P < 0.01). The asterisks above the middle set of arrows in the graphs indicate pairwise comparisons for the pre-treated and post-treated columns (Mann-Whitney U-test; *** P < 0.001; ** P < 0.01; * P < 0.05; ns, not significant). The grids below the graphs show pairwise comparisons (the compared columns are indicated with X) for the pre-treated or post-treated cells under different irradiation conditions (unpaired t-test; *** P < 0.001; ** P < 0.01; * P < 0.05; ns, not significant).

Figure S4. Distribution of the anti-γH2AX nuclear signal in cell populations following 48 h treatment of U-251 MG (a) or T98-G (b) cells with individual compounds and their mixtures in three independent experiments. Each coloured point in graph corresponds to a single detected nucleus; thick black line indicates median. EXP denotes independent experiment, L stands for lomustine and A for azacytidine.

Figure S5. Effect of dilution series of individual compounds and their mixture on U-251 MG cell number as determined by the IF assay. The graph shows pIC_50_ values ± standard errors of mean (N = 3) established from the normalized dose-response following 48 h treatment. The larger is the height of each column, the more efficient is the corresponding treatment. For the mixture, the molar ratio of components in each point of the dose-response curve is listed in brackets. The asterisks above the columns indicate pairwise comparisons for the individual compounds *vs* mixture (unpaired t-test; *** P < 0.001; ** P < 0.01); the same statistical significance parameters were obtained for the grouped analysis of the individual compounds *vs* mixture (1-way ANOVA with Dunnett’s test for multiple comparisons; *** P < 0.001; ** P < 0.01).

Figure S6. Representative microscopy images from IF assay in U-251 MG (left panel) and T98-G (right panel). The channels are indicated above the images and the treatment conditions (48 h) on the left. For better visualization, the brightness of images was increased by 40% and the contrast reduced by 40%; please note that the automated analysis of images was carried out using non-modified raw data. The images were collected within the same independent experiment. Scale bar (top left): 50 μm.

Figure S7. Example microscopy images from IF assay in U-251 MG featuring strong coloring in γH2AX channel following 48 h treatment of cells with 16.7 μM lomustine. The channels are indicated above the images. The images were collected within the same independent experiment. Scale bar (top left): 50 μm.

Supplementary Methods

*Chemicals and labware used for cell culturing, viability assay and fluorescence microscopy*

The solutions and growth medium components for the cell culture were obtained from the following sources: phosphate-buffered saline (PBS), fetal bovine serum (FBS) – Sigma-Aldrich (Steinheim, Germany); Eagle's Minimum Essential Medium (EMEM) modified to contain Earle's Balanced Salt Solution, non-essential amino acids, 2 mM L-glutamine, 1 mM sodium pyruvate, and 1500 mg/L sodium bicarbonate – ATCC (Manassas, VA, USA); a mixture of penicillin, streptomycin, and amphotericin B – Capricorn (Ebsdorfergrund, Germany). Resazurin and PBS for viability assay (supplemented with Ca^2+^, Mg^2+^) were from Sigma-Aldrich (St Louis, MO, USA).

The cells were grown at 37 °C in 5% CO_2_ humidified incubator (Sanyo; Osaka, Japan). For viability assay, the initial number of cells was counted using TC-10 cell counter (Bio-Rad; Hercules, CA, USA). For the dose-response studies of targeted compounds and their mixtures with chemotherapeutic agents, the cells were seeded onto transparent 96-well clear flat bottom cell culture plates BioLite 130188; for the radiation studies, 12-well clear flat bottom plates BioLite 130185 were used (both from Thermo Fischer Scientific; Rochester, NY, USA). For transfer of cell to irradiation facility, gas-permeable adhesive moisture seals were used (Brooks Life Sciences, Wotton, Surrey, UK). The plates were positioned between the 2 slabs of solid water phantom (30 cm × 30 cm × 5 cm below, 30 cm × 30 cm × 1 cm above the plates) and irradiated with doses 2 Gy or 4 Gy (Gantry angle 0°, collimator angle 0°, field size 25 cm × 25 cm, source to skin distance 100 cm, dose rate 0.6 Gy/min). Irradiation was performed at room temperature. Non-irradiated plates were also transported to the irradiation facility but were kept in the linear accelerator’s control room.

For microscopy, the cells were grown on 96-well Ibidi black μ-plates (ibidi GmbH, Gräfelfing, Germany). For fixation of cells, methanol was obtained from Honeywell (Riedel-de Haën™, Seelze, Germany). For preparation of washing and blocking solutions in IF experiments, Triton X-100 from AppliChem (Darmstadt, Germany), bovine serum albumin fraction V (BSA) from Capricorn Scientific (Ebsdorfergrund, Germany) and PBS (supplemented with Ca^2+^, Mg^2+^) from Sigma-Aldrich (Steinheim, Germany) were used.

*Viability assay*

The growth media was removed from the cells, the cells were rinsed with PBS, and 50 μM resazurin solution in PBS (containing Ca^2+^ and Mg^2+^) was applied onto the cells (final total volume of 100 μL in case of 96-well plates and 1 mL in case of 12-well plates). The plates were placed into multi-mode reader, and measurements were performed at 30 °C in kinetic mode (reading taken every 15 min for 2 h) using the following parameters: (A) fluorescence: excitation 540 (20) nm, emission 590 (20) nm, monochromator, top optics, gain 50; (B) absorbance at 570 nm and 600 nm, monochromator; read height 8.5 mm.

*Details of statistical analysis*

In each independent viability experiment, the fluorescence intensity measured for the replicate treatments was pooled and the data obtained for the negative control was plotted against incubation time with resazurin. One time-point within duration of data acquisition was chosen where the signal of the negative control remained in the linear range, and only data measured at this time-point was used for the further analysis. For normalization, data obtained for wells treated with PBS (blank control) was considered as 100% viability; data acquired for the 50 μM resazurin solution (in the absence of cells) was considered as 0% viability. Next, the ratio of absorbance at 570 nm and 600 nm was calculated for each well. The ratios were analyzed analogously to the fluorescence intensity data, and the normalized viability values calculated from the fluorescence intensity and the absorbance measurements were pooled. Finally, data from all independent experiments was pooled for each individual compound or mixture (N ≥ 4).

In case of dose-response studies of individual compounds and mixtures, the pooled normalized viability was plotted against the concentration of compound in the dilution series and fitted to the logarithmic dose-response function (three parameters). In case of mixtures, the concentration used on the X-axis corresponded to that of temozolomide or lomustine. The statistical significance of difference of calculated negative logarithms of IC_50_ values (pIC_50_) for the pairwise comparison of mixture *vs* the corresponding individual components was assessed using the unpaired two-tailed t-test with Welch’s correction (95% confidence level). Alternatively, a grouped comparison of individual components vs mixture was carried out using one-way ANOVA (95% confidence level) with Dunnett test for multiple comparisons.

In case of radiation experiments, the statistical significance of viability difference for the grouped comparisons (negative control on the non-irradiated plate *vs* all other conditions, or negative controls on the irradiated plates *vs* the other conditions on the same plates) was assessed using the one-way ANOVA (95% confidence level) with Dunnett test for multiple comparisons. For the various pairwise comparisons (pre-treatment *vs* post-treatment for different irradiation conditions, or pre-treatment for an irradiated *vs* non-irradiated plate), the unpaired two-tailed t-test with Welch’s correction or Mann-Whitney U-test (95% confidence level) was used.

In case of IF experiments, the data obtained from the automated image analysis was processed further as follows. For each independent experiment with 1 μM azacytidine and two different concentrations of lomustine (100 μM and 16.7 μM) in the presence or absence of 1 μM azacytidine, the mean γH2AX signal in nucleus was averaged for all nuclei in each treatment condition. The data for three independent experiments was then pooled and the statistical significance of γH2AX staining difference for the grouped comparisons (PBS control *vs* other conditions) was assessed using the one-way ANOVA (95% confidence level) with Dunnett test for multiple comparisons. For the pairwise comparisons (treatment with lomustine alone *vs* mixture), the unpaired two-tailed t-test with Welch’s correction or Mann-Whitney U-test (95% confidence level) was used. Subsequently, the number of nuclei identified in wells treated with PBS, 1 μM azacytidine, and 16.7 μM lomustine in the presence or absence of 1 μM azacytidine was normalized to that in PBS control for each independent experiment. The data for 3 independent experiments was then pooled and the statistical significance of cell number difference for the grouped comparisons (mixture-treated cells *vs* other conditions) was assessed using the one-way ANOVA (95% confidence level) with Dunnett test for multiple comparisons.

For microscopy experiments with dilution series of lomustine, azacytidine, and 1:1 mixture of lomustine with azacytidine, the number of nuclei was normalized for each replicate dilution series in each independent experiment (data obtained for wells treated with the lowest concentration of compounds was considered as 100% count). The data for all replicates in three independent experiments was then pooled and fitted to the logarithmic dose-response function (three parameters; bottom plateau constrained to 0% and upper plateau shared for all datasets). The statistical significance of difference of pIC_50_ values for the pairwise comparison of mixture *vs* the corresponding individual components was assessed using the unpaired two-tailed t-test with Welch’s correction (95% confidence level). Alternatively, a grouped comparison of individual components vs mixture was carried out using one-way ANOVA (95% confidence level) with Dunnett test for multiple comparisons.

Supplementary References

1. Dice, L. R. Measures of the Amount of Ecologic Association Between Species. *Ecology* **26**, 297–302 (1945).

2. Sørensen, T. J. *A method of establishing groups of equal amplitude in plant sociology based on similarity of species content and its application to analyses of the vegetation on Danish commons.* (I kommission hos E. Munksgaard, 1948).

3. Matthews, B. W. Comparison of the predicted and observed secondary structure of T4 phage lysozyme. *Biochimica et Biophysica Acta (BBA) - Protein Structure* **405**, 442–451 (1975).
